# Visualizing Protein-DNA Interactions in Live Bacterial Cells Using Photoactivated Single-molecule Tracking

**DOI:** 10.3791/51177

**Published:** 2014-03-10

**Authors:** Stephan Uphoff, David J. Sherratt, Achillefs N. Kapanidis

**Affiliations:** ^1^Microbiology Unit, Department of Biochemistry, University of Oxford; ^2^Biological Physics Research Group, Clarendon Laboratory, Department of Physics, University of Oxford

**Keywords:** Immunology, Issue 85, Super-resolution microscopy, single-particle tracking, Live-cell imaging, DNA-binding proteins, DNA repair, molecular diffusion

## Abstract

Protein-DNA interactions are at the heart of many fundamental cellular processes. For example, DNA replication, transcription, repair, and chromosome organization are governed by DNA-binding proteins that recognize specific DNA structures or sequences. *In vitro* experiments have helped to generate detailed models for the function of many types of DNA-binding proteins, yet, the exact mechanisms of these processes and their organization in the complex environment of the living cell remain far less understood. We recently introduced a method for quantifying DNA-repair activities in live *Escherichia coli* cells using Photoactivated Localization Microscopy (PALM) combined with single-molecule tracking. Our general approach identifies individual DNA-binding events by the change in the mobility of a single protein upon association with the chromosome. The fraction of bound molecules provides a direct quantitative measure for the protein activity and abundance of substrates or binding sites at the single-cell level. Here, we describe the concept of the method and demonstrate sample preparation, data acquisition, and data analysis procedures.

**Figure:**
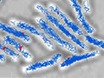


## Introduction

This protocol describes the direct measurement of protein-DNA interactions in living *Escherichia coli* cells. The technique utilizes the change in the diffusion coefficient of a single fluorescently labeled protein as it binds the chromosome (**Figure 1**). To demonstrate the method we use DNA polymerase I (Pol1), a prototypical DNA-binding protein that fills DNA gaps in lagging strand replication and excision repair pathways^1^.

The advent of super-resolution fluorescence microscopy enables visualization of molecular structures in cells with nanometer resolution. Photoactivated Localization Microscopy (PALM) employs fluorescent proteins that can be activated from an initial dark state to a fluorescent state (**Figure 2**). Only a subset of all labeled molecules is activated at any time to determine their positions in a sequential manner, independently of the total concentration of labeled molecules in the sample^2^. The localization precision per molecule mainly depends on the size of the fluorescent Point Spread Function (PSF), the number of collected photons, and the background signal^3^. Many applications of this method focus on the improved visualization of cellular structures. The realization that PALM can be combined with single-molecule tracking^4^ opened new avenues to directly follow the movement of arbitrary numbers of labeled proteins in living cells. Increased sensitivity and temporal resolution of fluorescence microscopes now allow tracking of single diffusing fluorescent proteins in the bacterial cytoplasm^5^.

Here, we employ PAmCherry, an engineered fluorescent protein that irreversibly converts from an initial nonfluorescent state to a fluorescent state upon irradiation with 405 nm light^6^. Activated PAmCherry fluorophores can be imaged by excitation at 561 nm and tracked for several frames until photobleaching. We demonstrate the ability of the method to identify transient DNA-binding events of single proteins using a fusion of Pol1 and PAmCherry. Treatment of cells with methyl methanesulfonate (MMS) causes DNA methylation damage that is turned into gapped DNA substrates by base-excision repair enzymes. Our method shows clear binding of single Pol1 molecules in response to MMS damage^7^.

## Protocol

### 1. Cell Culture

Use sterile culture tubes and pipette tips. The *E. coli* strain AB1157 *polA-PAmCherry* carries a C-terminal PAmCherry fusion of Pol1. The fusion was inserted at the native chromosomal location by replacing the wild-type gene using lambda-Red recombination as described in Datsenko *et al.*^8^ Functionality of the fusion protein was confirmed as judged by cellular growth rates and sensitivity to the DNA damaging agent methyl methanesulfonate (MMS). More information on construction of the cell strain can be found in Uphoff *et al.*^7^, Datsenko *et al.*^8^, and**Reyes-Lamothe *et al.*^9^ Cell cultures are grown in M9 minimal medium to reduce autofluorescence and avoid background particles on the microscope slide. Alternatively, a nutrient-rich defined medium can be used^10^.

1. Streak the *E. coli* strain AB1157 *polA-PAmCherry* from a frozen glycerol stock on a Luria Broth (LB) agarose plate with selective antibiotics (here, 25 µg/ml kanamycin) and incubate at 37 °C overnight.
2. Inoculate a 5 ml LB culture from a single cell colony and grow at 37 °C shaking at 220 rpm for 3 hr.
3. Dilute the culture 1:10,000 into 5 ml minimal medium (M9 medium, MEM amino acids + proline, MEM vitamins, 0.2% glycerol) and incubate at 37 °C shaking at 220 rpm overnight.
4. The following morning, measure the optical density (OD) using a spectrophotometer and dilute the culture in 5 ml fresh minimal medium to OD 0.025. Grow for 2 hr at 37 °C shaking at 220 rpm to early exponential phase (OD 0.1).
5. Concentrate 1 ml of cells in a 1.5 ml microcentrifuge tube by centrifugation at 2,300 x g for 5 min. Remove the supernatant and resuspend the cell pellet in 20 µl residual medium and vortex.

### 2. Microscope Slide Preparation

1. Prepare a 1.5% low-fluorescence agarose solution in dH_2_O. Use a microwave to melt the agarose until the solution is clear. Mix 500 µl of the melted agarose solution with 500 µl of 2x minimal medium by gently pipetting up and down a few times.
2. Spread the agarose solution evenly on the center of a microscope coverslip (No 1.5 thickness). This has to be done quickly before the agarose cools, avoiding bubbles.
3. Flatten the pad with a second coverslip (No 1.5 thickness). To remove background fluorescent particles, coverslips were previously burned in a furnace at 500 °C for 1 hr. Burned coverslips can be stored for weeks at room temperature covered in aluminum foil.
4. For DNA damage experiments, prepare an agarose pad containing 100 mM MMS. Follow the procedure in steps 2.1-2.3, but add 8.3 µl MMS to 500 µl of M9 medium before mixing with 500 µl of melted 1.5% agarose, for a final concentration of 100 mM MMS. (**Caution!** MMS is toxic and mutagenic and must be handled with gloves, mask, goggles, and lab coat).
5. Remove the top slide from the pad and add 1 µl of concentrated cell suspension onto the pad. Immobilize the cells by covering the pad with an unused burned coverslip (No 1.5 thickness, matching the microscope objective specification) and by pressing very gently on the slide. Cells should be imaged within 45 min of immobilization before the agarose pad dries. To prevent drying during longer experiments, agarose pads can be sealed using silicon gaskets.
6. For DNA-damage experiments, incubate cells immobilized on the agarose pad containing 100 mM MMS for 20 min in a humidified container at room temperature before imaging.

### 3. Preparing Microscopy Data Acquisition

PALM relies on the detection and precise localization of single fluorescent proteins. The sensitivity and optimal alignment of the microscope is critical for the data quality. Single-molecule fluorescence microscopes typically employ Total Internal Reflection (TIR) illumination to enhance the signal-to-noise ratio by exciting only fluorophores within a thin section above the coverslip surface. Here, imaging inside *E. coli* requires highly inclined illumination^11^, which can be achieved on a TIRF microscope by slightly decreasing the angle of the excitation light. PAmCherry imaging further requires a 405 nm photoactivation laser and a 561 nm excitation laser. The fluorescence emission is recorded on an electron multiplying CCD (EMCCD) camera at a magnification resulting in a pixel length of 114.5 nm/pixel. For optimal localization precision, the pixel size should approximately match the standard deviation width of the PSF to ensure sufficient sampling without spreading the signal over too many pixels. **Figure 3** shows a schematic of a minimal PALM setup. **Movie 1** gives an impression of the bespoke microscope building process; see Uphoff *et al*.^7^ for a detailed description of the instrument.

1. Performing routine microscope alignment. Measure the 405 nm and 561 nm continuous wave laser intensities in front of the objective. Adjust the 561 nm intensity to 3.5 mW (~400 W/cm^2^) and 405 nm intensity to 10 µW (~1 W/cm^2^). Use a continuously variable neutral density filter wheel that allows gradual adjustment of 405 nm intensity from 0-10 µW. Switch off the laser illumination until the start of the experiment.
2. Place the sample on the microscope stage and bring the cells into focus in transmitted light microscopy mode (**Figure 4A**). The EMCCD camera gain has to be switched off to prevent damage to the camera by overexposure.
3. Define a cropped FOV to reduce data size and increase the camera read-out speed.
4. Cover the sample from ambient light and switch on the EMCCD camera gain.
5. Set the frame rate to 15.26 msec/frame (including 0.26 msec camera readout time). See "Exposure time and excitation intensities" in the Discussion section.
6. Display the camera data to check the dark background signal (**Figure 4B**).
7. Switch on the 561 nm laser and check the excitation background signal (**Figure 4C**).
8. Switch on the 405 nm laser for photoactivation of the Pol1-PAmCherry fusion proteins and increase the intensity until fluorescence PSFs appear.
9. Adjust the angle of the excitation beam to illuminate only a thin section of the sample close to the coverslip surface.
  1. To this end, the laser beam is focused into the back focal plane of a 100X NA 1.4 objective (**Figure 3**). Translating the focusing lens perpendicular to the beam moves the focus away from the center of the objective causing the beam to exit the objective under an angle.
  2. Aim to maximize the fluorescence intensity and minimize the background signal. Note that strict TIR excitation is optimal to image fluorophores within 100 nm of the coverslip surface, however, imaging DNA-binding proteins associated with the *E. coli* nucleoid requires deeper illumination up to 0.8 µm.

### 4. Data Acquisition

Here, we describe the general protocol for acquisition of a PALM movie. The same procedure applies for imaging Pol1-PAmCherry fusion proteins in undamaged *E. coli* cells and under continuous DNA damage treatment with MMS. Application of the method to fusion proteins of different molecular weight or copy number per cell will require different acquisition settings (see Discussion section).

1. Find a new field of view (FOV) of cells in transmitted light microscopy mode and focus the image. Take a camera snapshot to record the cell outlines (**Figure 4A**).
2. Cover the sample from ambient light and switch on the EMCCD camera gain.
3. Switch on the 561 nm laser and bleach the cellular autofluorescence and background spots on the coverslip for a few seconds before starting data acquisition. For cells grown and imaged in M9 medium and using burned coverslips there is usually very little fluorescence background; however, prebleaching could be useful for imaging cells in a rich growth medium such as LB. Note that intense illumination is toxic to cells so prebleaching should be kept to a minimum.
4. Start the acquisition of a PALM movie under continuous 561 nm excitation at 15.26 msec/frame.
5. Switch on the 405 nm laser and gradually increase the intensity over the course of the movie, reaching up to 1 W/cm^2^. Avoid higher 405 nm intensities that cause cellular autofluorescence. Pay attention to the density of fluorescent molecules - it is important to keep activation rates low such that PSFs are clearly isolated in each frame (**Figures 4D-F**).
6. Record 10,000 frames/movie (depending on the number of molecules to be imaged per cell); one movie typically takes 2-3 min and requires 0.5-1 GB of hard disk space depending on the size of the FOV.
7. Repeat the acquisition procedure for multiple FOV. Note that each FOV can only be imaged once because PAmCherry fluorophores get photoactivated and bleached irreversibly.

### 5. Data Analysis

An automated and robust data analysis framework is essential for the performance and efficiency of the method. We use custom software written in MATLAB.

1. Perform the localization analysis using algorithms described in Crocker *et al.*^12^, Holden *et al.*^13^, HoldenI *et al*.^14^, and Wieser *et al.*^15^ PSFs are first identified in a band-pass filtered image using a Gaussian kernel with 7 pixels diameter (**Figure 5A**). Candidate positions correspond to PSFs with peak pixel intensities above 4.5 times the standard deviation of the background signal (**Figure 5B**). The locally brightest pixel per candidate PSF serves as initial guess for fitting an elliptical Gaussian function (**Figure 5C**). The free fit parameters are: x-position, y-position, x-width, y-width, rotation angle, amplitude, and background offset. The elliptical Gaussian mask accounts for molecule during the exposure time, which blurs and deforms the PSF.
2. Plot the resulting (x, y) localizations from all frames of the PALM movie onto the transmitted light microscopy image of the same FOV. Localizations of Pol1-PAmCherry should appear within the central area of *E. coli* cells (**Figure 6A**). If many localizations appear outside of cells, the localization threshold was set too low or the sample contained background fluorescent particles.
3. For automated tracking analysis, the MATLAB implementation of the algorithm described in Crocker *et al.*^12^ can be used (see "Diffusion analysis" in the Discussion section). Positions that appear in subsequent frames within a user-defined tracking window are connected to form a trajectory. In the case that multiple localizations occur in the same window, tracks are uniquely assigned by minimizing the sum of step lengths. For a detailed discussion of the various considerations when calculating diffusion coefficients from single-particle tracking data, see Wieser *et al.*^15^
  1. The algorithm uses a memory parameter to account for transient blinking or missed localizations during a track. Here, we set the memory parameter to 1 frame; higher values can be used for tracking fluorophores with long-lived dark states.
  2. Choose a suitable tracking window based on the following calibration steps. For Pol1, we use 0.57 µm (5 pixels).
  3. Run the tracking algorithm for a range of tracking window parameters. Calculate the number of measured tracks per cell as a function of the tracking window to identify the smallest possible tracking window that does not split tracks (**Figure 6B**).
  4. Plot the resulting tracks on the transmitted light microscopy image of the same FOV to visualize the spatial distribution of molecule movement within cells. Pol1 tracks should display diffusion confined within single cells (**Figures 6C-D**).
  5. If a fraction of tracks appears to cross between cells this suggests that separate molecules were erroneously linked because the tracking window was chosen too large and/or the photoactivation rate was too high (**Figure 6E**).
  6. Plot the cumulative distribution of the step lengths between consecutive localizations (**Figure 6F**). The curve rises and saturates smoothly for sufficiently large tracking windows but shows a cutoff edge if the window was chosen too small.
4. To analyze the diffusion characteristics of Pol1, compute the mean-squared displacement (MSD) between consecutive localizations for each track with a total of N steps): MSD = 1/(N-1) ∑ _i = 1 _^N-1^(x_i+1 _– x_i_)^2 ^+ (y_i+1 _– y_i_)^2^ . Include only tracks with at least 4 steps (N ≥ 5 localizations) to reduce the statistical uncertainty in the MSD values.
5. Plot a curve of MSD values over a range of lag times by calculating displacements over multiple frames (**Figure 6G**). The shape of the MSD curve can help to classify the observed molecular motion (**Figure 6H**).
6. Calculate the apparent diffusion coefficient D* per track from the MSD: D* = MSD/(4 Δt) – σ_loc_^2^/Δt . The second term corrects for the estimated localization error (here, σ_loc _= 40 nm and Δt = 15.26 msec, see Wieser *et al.*^15^).
7. Plot a histogram of the measured D* values from all tracks in the FOV (**Figure 7A**).
8. Identify individual Pol1 molecules that appear bound to the chromosome based on the measured D* value per track. Separate the populations of bound (sharp distribution centered at D* ~ 0 µm^2^/sec) and freely diffusing molecules (broad distribution centered at D* ~ 0.9 µm^2^/sec) by setting a threshold D* < 0.15 µm^2^/sec (red bars in **Figures 7A **and** 7D**).
9. Carry out the localization, tracking, and diffusion analysis for Pol1 in undamaged cells (**Figures 7A-C**) and in cells under DNA-damage treatment with MMS (**Figures 7D-E**). The fraction of bound tracks provides a direct quantitative measure of the DNA repair activity of Pol1 *in vivo*.

## Representative Results

The concept of photoactivated single-molecule tracking to study protein-DNA interactions *in vivo* is illustrated in **Figure 1**. PAmCherry fusion proteins are detected in live *E. coli* cells in a sequential manner by photoactivating single molecules stochastically with 405 nm light at a frequency of less than one molecule per cell at a time. Activated molecules are imaged under continuous 561 nm excitation. Molecular movement in the cell can be tracked by connecting nearby localizations in a series of frames until irreversible photobleaching. Because the diffusion of DNA-binding proteins is slowed upon binding the chromosome, the apparent diffusion coefficient D* obtained per track directly reports on individual protein-DNA interactions.

**Figure 2** demonstrates photoactivation of Pol1-PAmCherry fusion proteins in live *E. coli* cells. The influence of the 405 nm intensity on the density of fluorescent molecules can be seen in **Figure 4**. Note that the density is not solely determined by the 405 nm intensity but additionally by the number of molecules that are available for activation; the pool of remaining molecules is depleted over the course of a PALM movie.

Localization analysis is performed for each frame of a PALM movie as illustrated in **Figure 5**. We measured the localization precision using immobile molecules in fixed cells or bound molecules in live cells. Our acquisition settings gave a localization precision of σ_loc_ = 40 nm, in agreement with the theoretical prediction^3^.

The resulting Pol1 localizations occupy the central area of the cell (**Figure 6A**), broadly recapitulating the spatial organization of the *E. coli* nucleoid^7^. The majority of Pol1 tracks in undamaged cells display diffusion as shown in **Figure 6C**. A typical cell contains several hundred Pol1 tracks (**Figure 6D**), consistent with the copy number of approximately 400 Pol1 molecules per *E. coli* cell^1^. **Figures 6B **and** 6E-F** provide guidance on choosing a suitable tracking window parameter – if the tracking window is too large, different molecules are more likely to become erroneously linked to a track; if the tracking window is too small, tracks with longer steps will be split. The MSD curve for Pol1 rises linearly for short lag times and saturates at longer lag times due to cell confinement (**Figure 6G**). Different types of molecular motion can be identified by MSD analysis. Directed motion gives a parabolic curve; Brownian motion is characterized by a straight line; the confined diffusion curve reaches a plateau; an offset of the MSD curve for immobile particles represents the localization uncertainty (**Figure 6H**). Additional information on single-particle tracking and troubleshooting tips can be found in Arnauld *et al*.^16^

We previously applied the method to measure the DNA-repair activity of Pol1 in response to exogenous DNA alkylation damage^7^. The D* histogram of Pol1 tracks in undamaged cells shows a dominant population of diffusing molecules (**Figures 7A-C**). A small fraction of 2.7% bound Pol1 molecules is likely involved in lagging strand replication and repair of endogenous DNA damage. Under continuous 100 mM MMS damage, the population of tracks with D* ~ 0 µm^2^/sec increases to 13.8% (**Figure 7D**). These tracks represent individual Pol1 molecules performing DNA repair synthesis to fill single-nucleotide gaps as part of the base-excision repair pathway. The positions of bound tracks show the locations of individual DNA damage and repair sites (**Figure 7E-F**).


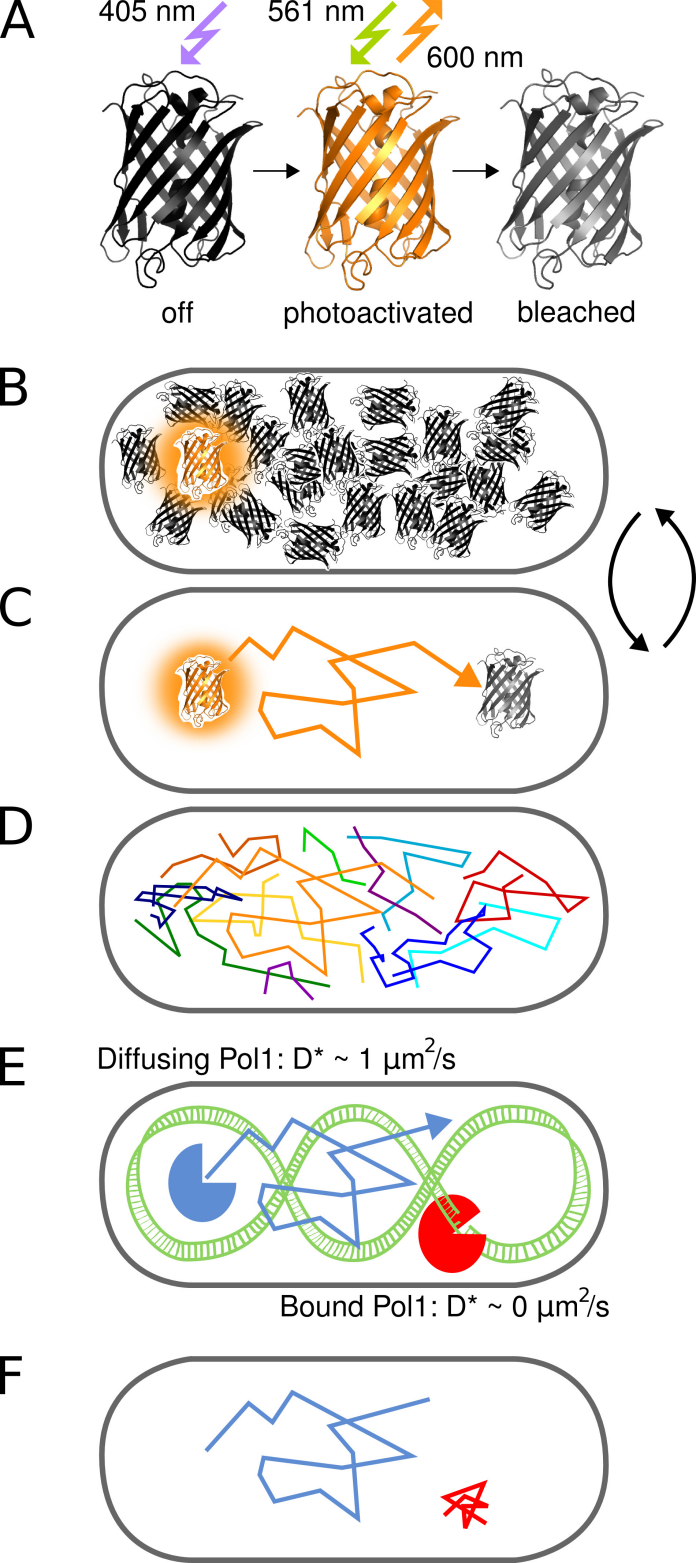
**Figure 1. Graphical representation of the method. **(**A**) The fluorescent protein PAmCherry can be photoactivated from an initial nonfluorescent state upon irradiation with 405 nm light. The bright state is excited at 561 nm and emits fluorescence around 600 nm until the fluorophore bleaches irreversibly. (**B**) Controlling the photoactivation rate allows imaging only a single stochastically activated PAmCherry fusion protein per cell at any time while the arbitrarily large pool of molecules that have not yet been activated or have already been bleached remains in a dark state. (**C**) The position of the fluorescent molecule is determined from the center of the isolated PSF and tracked for several frames until photobleaching. (**D**) Tracks of many molecules are recorded in a sequential manner. (**E-F**) The interaction of a DNA-binding protein with a chromosomal target sequence or structure halts the random diffusive motion. Bound and unbound molecules are distinguished by the apparent diffusion coefficient D* extracted from single tracks. The resulting fraction of bound molecules gives a quantitative measure for the activity of a DNA-binding protein *in vivo*. Click here to view larger image.


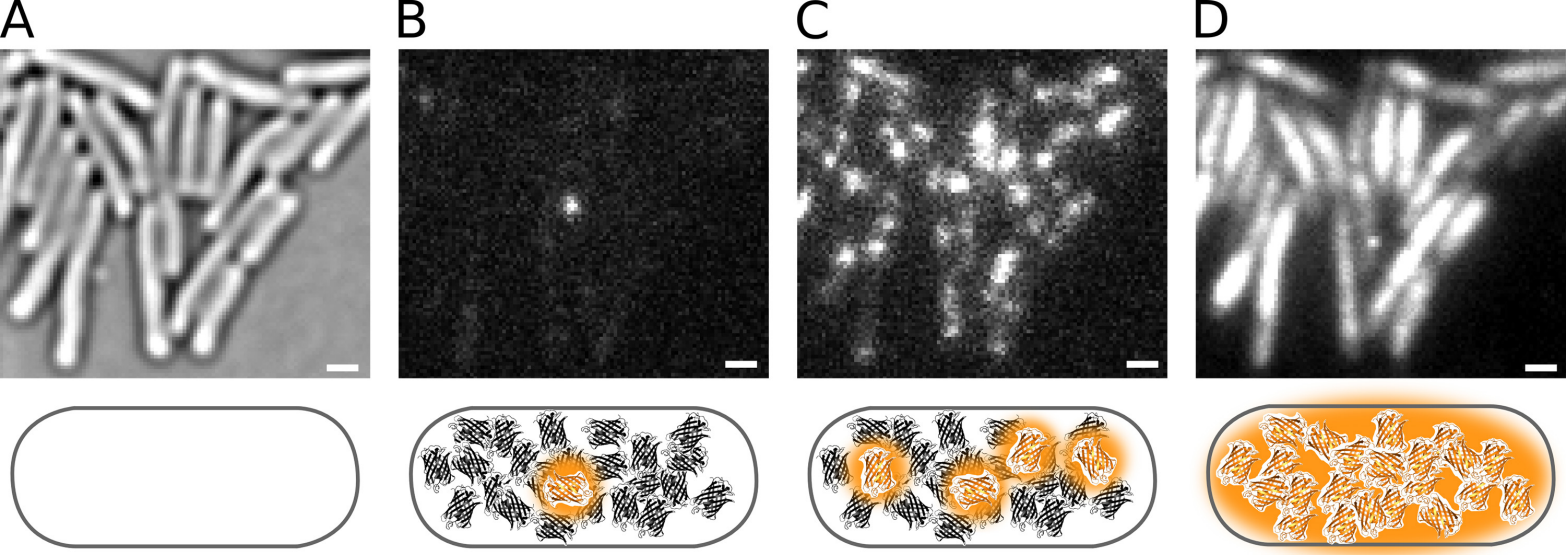
**Figure 2. Photoactivation of Pol1-PAmCherry in live *E. coli* cells**. Scale bars: 1 µm. Schematics are shown underneath each panel. (**A**) Transmitted light microscopy image of cells immobilized on an agarose pad. (**B**) Phototactivating a single PAmCherry fluorophore in one cell. (**C**) A higher photoactivation rate increases the number of fluorescent molecules. (**D**) Integrated PAmCherry fluorescence from a PALM movie. Click here to view larger image.


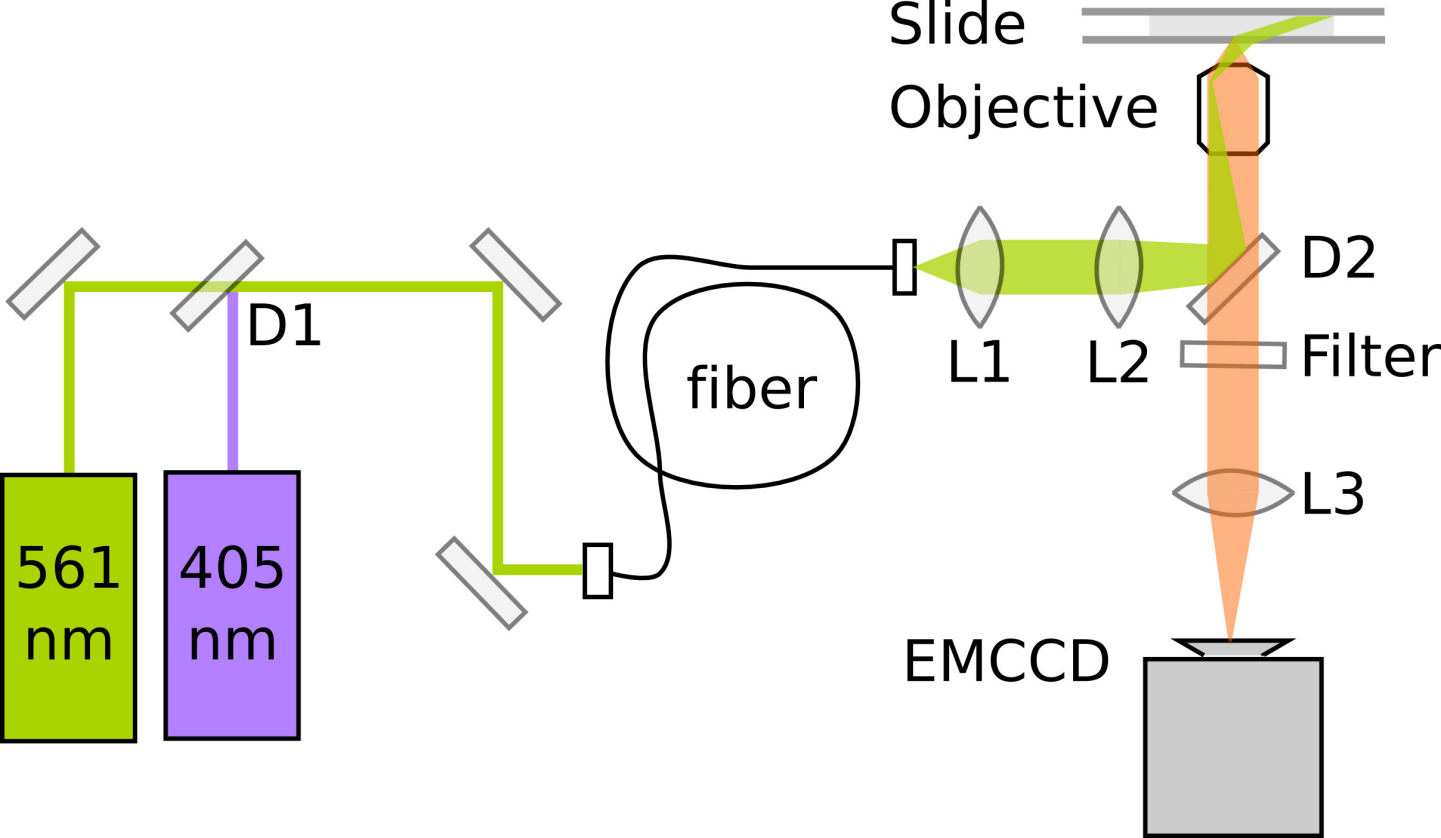
**Figure 3. Schematic of a minimal PALM setup for photoactivating and imaging PAmCherry fusion proteins.** D1: Dichroic mirror (*e.g.* 550 nm long-pass). D2: Dichroic mirror (*e.g.* 570 nm long-pass). L1: Collimating lens. L2: TIR lens. L3: Tube lens. Click here to view larger image.


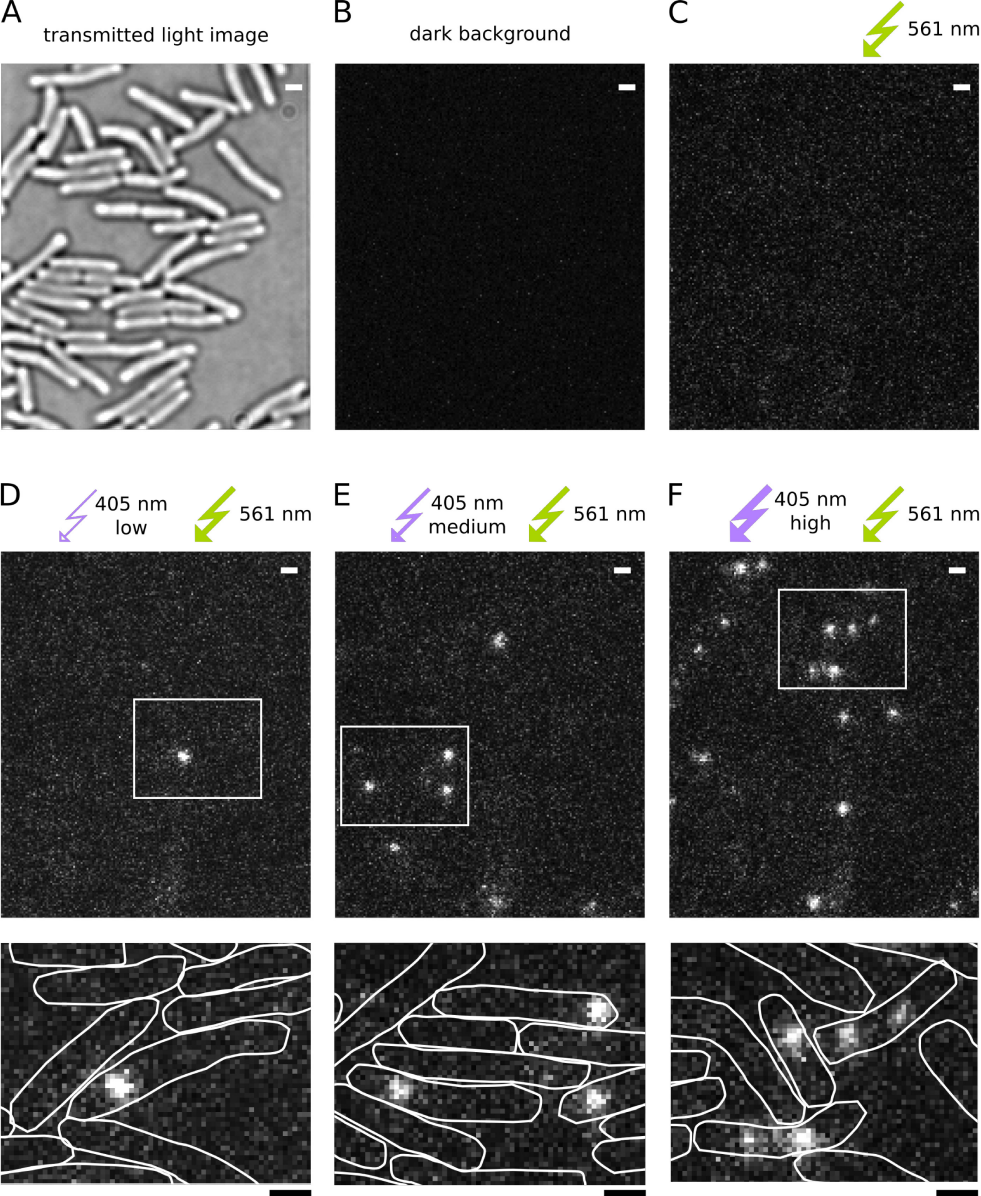
**Figure 4. Representative images from a PALM movie with 15.26 msec/frame.** Scale bars: 1 µm. (**A**) Transmitted light image of cells immobilized on an agarose pad. (**B**) Dark background image measured on the EMCCD camera with the lasers switched off. (**C**) Excitation background image under continuous 561 nm excitation before photoactivation. (**D-F**) Increased 405 nm intensity leads to higher photoactivation rates of PAmCherry, imaged under continuous 561 nm excitation. The boxed areas are shown magnified below. (**D**) Low 405 nm intensity (<1 µW) actives very few fluorescent molecules per FOV. (**E**) Medium 405 nm intensity (~2 µW) photoactivation results in a good PSF density for localization and tracking analysis. (**F**) Higher 405 nm intensity (~10 µW) activates more than one fluorescent molecule in some cells, which obscures localization and tracking analysis. Click here to view larger image.


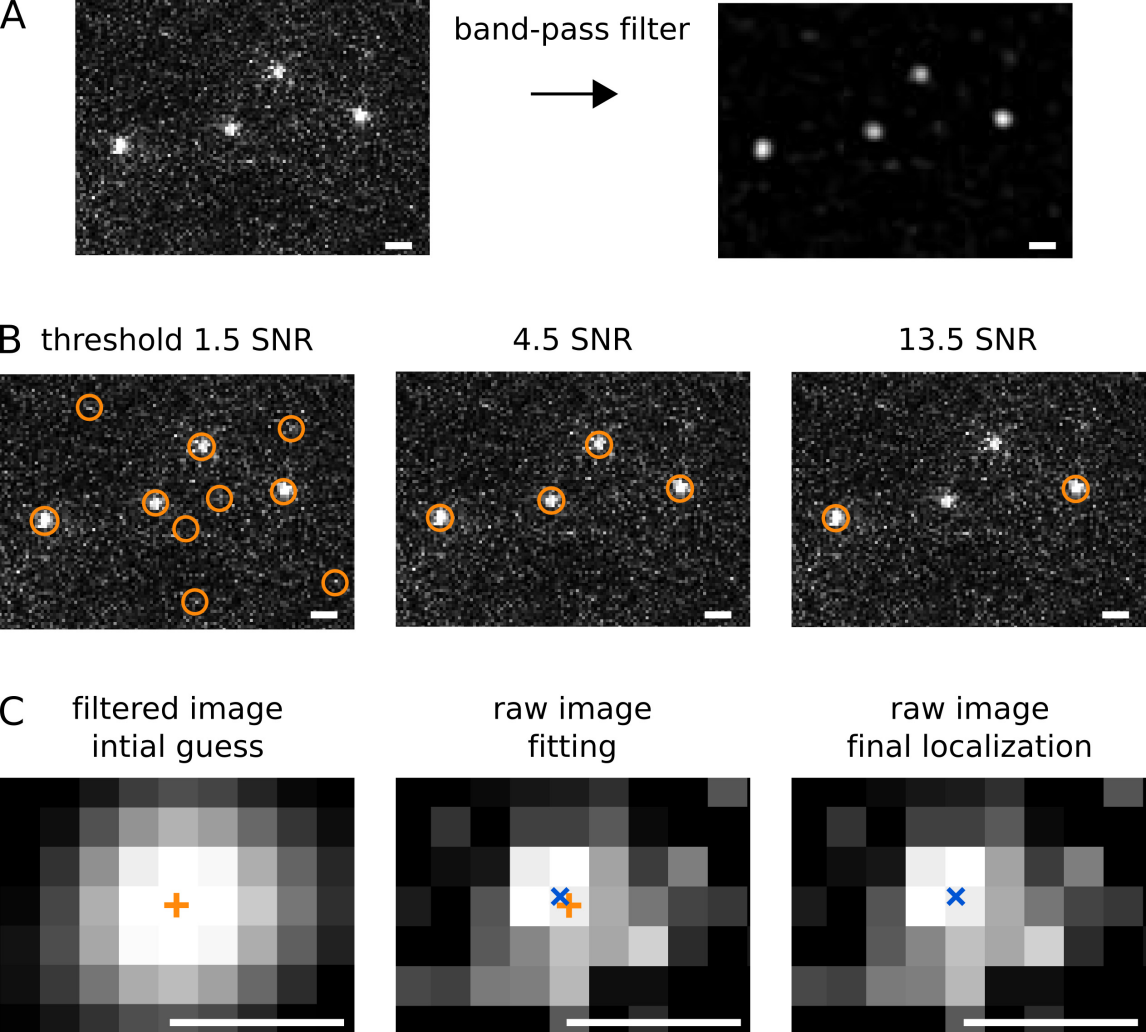
**Figure 5. Illustration of the localization analysis. Scale bars: 1 µm.** (**A**) Band-pass filtering removes spurious pixel noise and flattens intensity gradients across the FOV. (**B**) Candidate PSFs are identified in the filtered image based on a user-defined threshold that is chosen to minimize false positive and false negative detections. The threshold corresponds to the minimum intensity of a candidate pixel divided by the background standard deviation (signal-to-noise ratio, SNR). (**C**) The locally brightest pixel that passes the threshold serves as initial localization guess (orange cross) for a two-dimensional elliptical Gaussian fit. Scale bars: 0.5 µm. The resulting super-resolution localization (blue cross) has an average precision of σ_loc_ = 40 nm. Click here to view larger image.


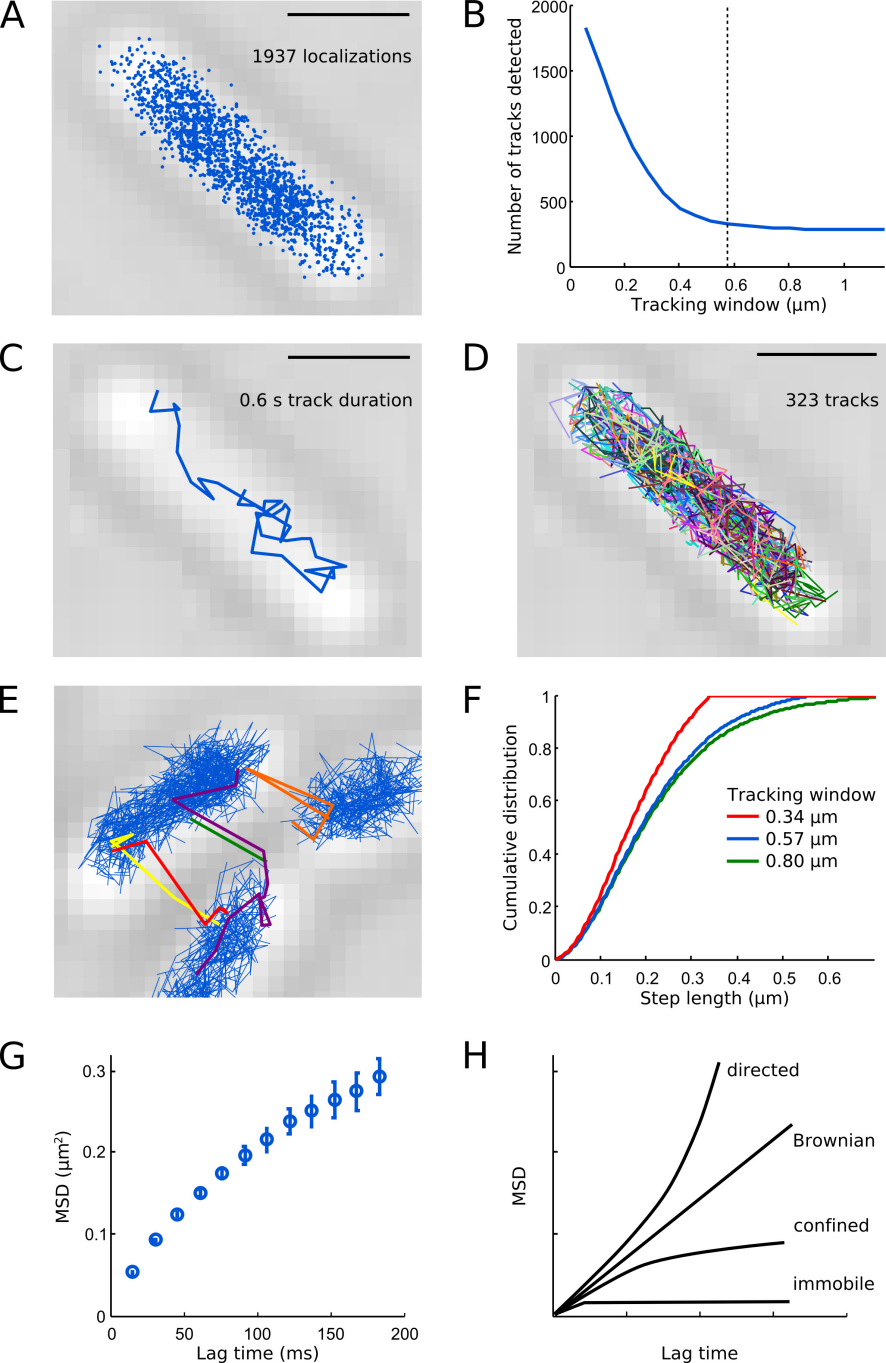
**Figure 6.** **Illustration of the tracking analysis. **Scale bars: 1 µm. (**A**) All detected localizations of Pol1-PAmCherry in an example cell. (**B**) Number of tracks detected in the example cell as a function of the tracking window. Small tracking windows split molecule trajectories, which leads to artifactual high number of tracks. The dashed line indicates our choice for the tracking window parameter (0.57 µm, 5 pixels) - this gives a good compromise between detecting the full distribution of steps and keeping the trajectories of different molecules intact. (**C**) Example track of a single Pol1-PAmCherry molecule. (**D**) All measured tracks shown in random colors. (**E**) Tracking artifacts if the tracking window is chosen too large (here 0.8 µm, 7 pixels) or the density of PSFs per frame is too high. (**F**) Cumulative distributions of the step lengths for tracking windows: 0.34 µm (3 pixels, red line), 0.57 µm (5 pixels, blue line), and 0.80 µm (7 pixels, green line). Note that the 0.34 µm tracking window cuts off steps longer than 0.34 µm which clearly truncates the full distribution of steps. The 0.57 µm tracking window detects almost the same distribution of steps as does the 0.80 µm tracking window. (**G**) MSD curve shows confined diffusion of Pol1. (**H**) Schematic MSD curves for directed motion, Brownian motion, confined diffusion, and immobile particles. Click here to view larger image.


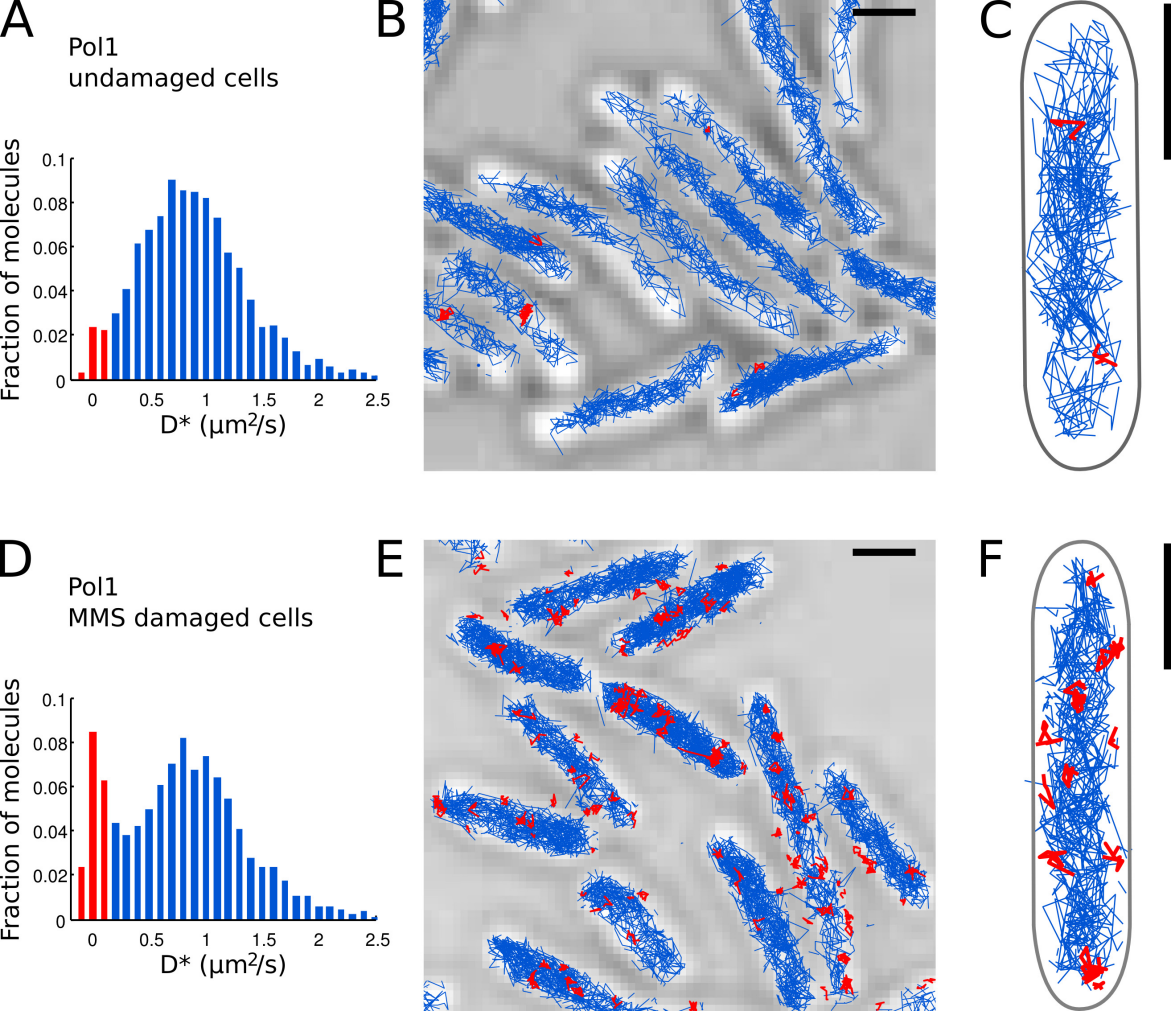
**Figure 7. Direct measurement of the DNA repair activity of Pol1 in live *E. coli* cells.** Scale bars: 1 µm. (**A**) Histogram of the apparent diffusion coefficient D* for all tracks of 4 or more steps in a FOV of undamaged cells (N = 4,162 tracks). The population of molecules classified as bound is highlighted in red. (**B-C**) Tracks of Pol1-PAmCherry are shown on a transmitted light microscopy image. Tracks classified as bound according to their diffusion coefficient are shown in red. (**D**) D* histogram for Pol1 tracks measured in cells immobilized on an agarose pad with 100 mM MMS and incubated for 20 min before imaging (N = 2,128 tracks). The population of bound molecules engaged in DNA repair is shown in red. (**E-F**) Pol1-PAmCherry tracks on transmitted light microscopy image showing the tracks of single Pol1 DNA repair events in red. Click here to view larger image.


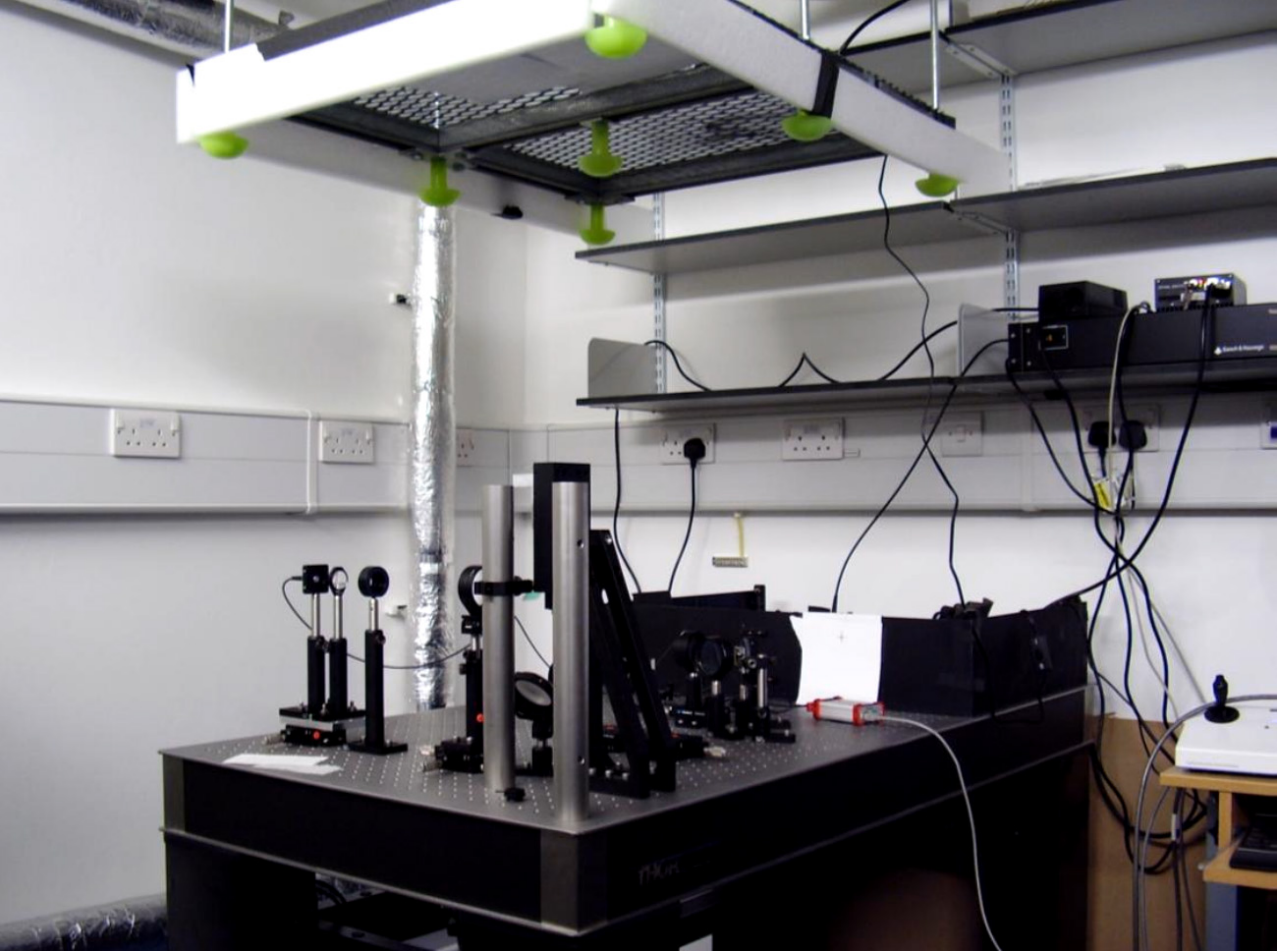
**Movie 1. Building a bespoke PALM setup**. Click here to view video.

## Discussion

We discuss several key considerations for the success of the experiment.

**Choice and expression of the fluorescent fusion protein:** There is a large palette of photoactivatable and photoswitchable fluorescent proteins^17^. The specific choice depends on the microscope characteristics, particularly the lasers and filters available. The combination of 405 nm and 561 nm is ideal for common photoactivatable fluorescent proteins. We chose PAmCherry^6^ because it is monomeric and showed no aggregation in cells. Furthermore, irreversible photoactivation allows counting the number of activated fluorophores to measure protein copy numbers per cell. Instead of expressing the fusion protein from a plasmid, we prefer chromosomal insertion of the gene encoding for the fusion protein at the wild-type locus. This ensures complete replacement of the protein of interest with the fluorescent version and maintaining the wild-type expression level.

**Photoactivation rate:** It is important to adjust the photoactivation rate such that on average less than one molecule per cell is in the fluorescent state in any frame of the movie. This depends on the 405 nm intensity and the number of molecules left to be activated. At very low imaging densities, however, not all molecules will be imaged before the end of the movie or very long movies have to be acquired. The number of frames recorded per movie depends on the copy number of fusion proteins per cell and the mean photobleaching lifetime of PAmCherry at the excitation conditions used. The copy number of Pol1 is ~400 molecules/cell^1^ and the mean value of the exponential photobleaching lifetime distribution was ~4 frames. By increasing the 405 nm intensity gradually, the activation is evenly distributed over the 10,000 frames of the movie. Therefore, each cell is occupied by fluorescent molecules for a total of ~1,600 frames, ensuring little overlap of PSFs and tracking complications in a movie of 10,000 frames.

**Exposure time and excitation intensities:** Foremost, exposure times need to be sufficiently short to observe sharp PSFs with little motion blurring. However, the frame rate should be chosen to yield observable molecular motion between successive frames beyond the localization uncertainty; otherwise crucial photons are wasted by oversampling the track. The motion of unbound molecules must be sampled at sufficiently long time intervals to be clearly distinguishable from the apparent motion of bound molecules due to the localization uncertainty. When the exposure time is set, the PSF intensity should be adjusted. The localization precision of a PSF increases with the number of photons detected over the duration of a frame. Higher excitation intensities increase the photon emission rate but also the photobleaching rate and background signal. Use the lowest excitation intensity that gives the desired localization precision. For Pol1-PAmCherry we chose 15.26 msec/frame and 3.5 mW 561 nm excitation (400 W/cm^2^). It is important to confirm cell viability for the particular imaging conditions by monitoring cell growth and morphology before and after data acquisition (see Supplementary Information in Uphoff *et al*.^7^).

Pol1 exhibits a binding time of ~2 sec to a gapped DNA substrate *in vivo*^7^; we therefore expect the majority of molecules to be either in the bound or unbound state for the whole duration of a track. Bound molecules appear essentially immobile because chromosome sites have a diffusion coefficient several orders of magnitude lower (~10^-5^ µm^2^/sec, Elmore *et al*.^18^) than Pol1 diffusion in the cytoplasm (~1 µm^2^/sec).

**Diffusion analysis:** The apparent diffusion coefficient D* is calculated from the MSD of individual tracks, averaged over a minimum of 4 steps (5 frames) to reduce the statistical error. Note that ~75% of molecules bleach within less than 5 frames for the imaging conditions described. Such short tracks do not provide sufficient statistical certainty to distinguish bound and unbound molecules. However, the relative fractions of bound and unbound molecules that report on the protein activity are independent of the total number of tracks analyzed.

It is useful to account for the PSF localization error (σ_loc_) in the calculation of D* because the uncertainty adds an apparent random step to each localization of a molecule^15^.

To improve the classification of bound and diffusing molecules, we recommend calculating D* both from the single-step displacements and the displacements over the time of two frames. It is then possible to set two separate D* thresholds: D*(15 msec) < 0.15 µm^2^/sec and D*(30 msec) < 0.075 µm^2^/sec.

Note that D* is an apparent diffusion coefficient that is affected by cell confinement of the tracks and motion blurring due to diffusion during the exposure time. To extract accurate unbiased diffusion coefficients, it has proven useful to compare the observed motion to simulated data based on a stochastic Brownian motion model^5,7^. Simulated data can also be used to test data analysis procedures.

**Potential applications of this method:** We described a general approach for visualizing and quantifying protein-DNA interactions *in vivo* by the change in the mobility of a protein upon binding to the chromosome. The activities of DNA- or RNA-binding proteins involved in repair, replication, transcription, and chromosome maintenance can thus be followed in real-time at the single-cell level with a spatial resolution below the optical diffraction limit. Photoactivated single-molecule tracking extends conventional tracking methods that are restricted to a few labeled molecules per cell. An alternative method that measures molecular diffusion *in vivo *is Fluorescence Recovery After Photobleaching (FRAP). While FRAP is very useful for measuring global diffusion characteristics in large cells, it is limited in its ability to resolve several molecular species with different mobilities in a spatially heterogeneous environment, especially for small bacterial cells.

We have applied photoactivated single-molecule tracking to measure DNA-binding activities and subcellular localizations of a range of different proteins in *E. coli* including Pol1, DNA ligase, Fis protein, DNA polymerase III^7^, as well as Structural Maintenance of Chromosomes proteins MukB, E, and F^19^. We anticipate the method can also be adapted to other cell types.

## Disclosures

The authors declare they have no competing financial interests.
